# Curette‐Assisted Removal of a Bronchial Screw Foreign Body Refractory to Forceps Grasping

**DOI:** 10.1002/rcr2.70642

**Published:** 2026-06-16

**Authors:** Genki Inui, Kohei Yamane, Masaki Nakamoto, Shino Arita, Shiro Moriyama, Takafumi Nonaka, Yasuhiko Teruya, Masaaki Yanai, Tomohiro Sakamoto, Naoki Kinoshita, Kosuke Yamaguchi, Masato Morita, Masahiro Kodani, Akira Yamasaki

**Affiliations:** ^1^ Division of Respiratory Medicine and Rheumatology, Department of Multidisciplinary Internal Medicine, Faculty of Medicine Tottori University Yonago Japan; ^2^ Division of Infectious Diseases, School of Medicine, Faculty of Medicine Tottori University Yonago Japan; ^3^ Cancer Center, Tottori University Hospital Yonago Japan

**Keywords:** Curette, endobronchial removal, foreign body, screw

## Abstract

Although forceps and snares are commonly used for bronchial foreign body removal, secure capture may be difficult in a narrow peripheral airway or when the object has a smooth surface. A curette may help mobilize such foreign bodies and enable subsequent extraction with forceps.

## Graphical abstract

A 70‐year‐old man with schizophrenia was found to have a screw lodged in the peripheral left B10c bronchus. Forceps and snare capture failed because of the screw's smooth surface and the narrow operative field. A curette was advanced beyond the screw to displace it centrally, enabling successful forceps extraction.

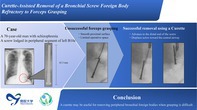

A 70‐year‐old man with long‐standing schizophrenia was incidentally found on chest computed tomography to have a screw lodged in the peripheral left B10c bronchus. Flexible bronchoscopic removal was attempted; however, forceps and snare capture failed because of the screw's smooth surface and the narrow operative field. A curette was advanced beyond the screw and used to displace it centrally, enabling successful extraction with forceps, as shown in Video 1. Few similar cases have been reported [1, 2]. This case demonstrates that a curette may facilitate removal of peripheral bronchial foreign bodies when conventional grasping is difficult.

**VIDEO 1:** video of the procedure. Video content can be viewed at https://onlinelibrary.wiley.com/doi/10.1002/rcr2.70642.

## Author Contributions

G.I. designed the study, prepared all the figures and videos, and wrote the manuscript. K. Yamane and M.K. performed the bronchoscopy and data collection. M.N., S.A., S.M., T.N., Y.T., M.Y., T.S., N.K., K. Yamaguchi, M.M., and A.Y. advised on the study design and contributed to the discussion and development of the study. All the authors reviewed the manuscript.

## Funding

The authors have nothing to report.

## Consent

The authors declare that written informed consent was obtained from the patient's family after the patient's death for the publication of this manuscript and accompanying images/videos using the form provided by the Journal.

## Conflicts of Interest

The authors declare no conflicts of interest.

## Data Availability

The data that supports the findings of this study are available in the article.
